# Supplementary material for: Case-control study of activities associated with SARS-CoV-2 infection in an adult unvaccinated population and overview of societal COVID-19 epidemic counter measures in Denmark
Source: PLoS One. 2022 Nov 16;17(11):e0268849. doi: 10.1371/journal.pone.0268849 (PMC9668151; doi:10.1371/journal.pone.0268849)
Supplement: Attachment — Submitted filename: Response to Reviewers_.docx [file pone.0268849.s001.docx]

Reviewer #1: The study is a case-control study done in Denmark to elucidate factors associated with SARS-CoV-2 infection. Findings from this paper may be context-specific (in the setting of the situation in Denmark, in the setting of the alpha-dominant period, etc.). Still, the study is methodologically sound and provides important insights into controlling SARS-CoV-2. There are several points the authors should address. A couple of other minor points suggested to improve the paper further are also included.

1. The author’s conclusion in the abstract and the main text should be modified so that it is directly supported by the data. Specifically, the following statement is not supported directly by the data: “Transmission of disease through involvement in community activities appeared to occur only rarely, suggesting that community restrictions in place were efficient.” According to Table 2, at least based on the self-reported place of infection, over half of the cases are infected in the community activities. Also, even if the community restrictions in place were efficient, behaviors that are truly high-risk would have been identified as such in the present study design.

**Answer:** Thank you for this comment. The abstract and the conclusion in the main text have been modified, so the conclusion is supported by our results. We have included that, as we found no single determination to be associated with SARS-CoV-2 infection and because transmission appeared to primarily take place via contact to a person with known SARS-CoV-2 infection, community restrictions in place were adequate to reduce risk to a level where we could not find a difference between cases and controls.

Abstract (Lines 20-23): Alcohol consumption and fitness centers were weakly associated with infection, in agreement with findings of our similar study conducted six month earlier. Apart from the two factors, no community activities were more common amongst cases, suggesting that community restrictions in place were adequate. The strongest risk factor for transmission was contact to an infected person.

Conclusion (Line 296-304): In conclusion, we show results of a study of risk factors for SARS-CoV-2 infection and compare with a similar study done six month earlier. Under the constraints of the methodology of case-control studies, no major determinants for infection were identified. Since no determinants for infection were identified, we conclude that overall societal restrictions in use in the spring of 2021 were adequate to reducing any potential risk associated with community activities such as participating in cultural events, dining at restaurants, shopping and public transportation; exceptions being fitness centers and alcohol consumption. Instead, a major risk factor for transmission was contact to an infected person and transmission primarily took place via infected colleagues or family members. Finally, we provide a timeline of non-pharmaceutical interventions that were implemented in Denmark from February 2020 to March 2022.

2. In the abstract, “confined space” and “contact to known persons” should be separated. “Contact to known persons” should be reworded to “contact to infected individuals.”

**Answer:** Thank you for this. The abstract has been rephrased. Confined space is deleted and “Contact to known persons” is reworded to “contact to a person with known SARS-CoV-2 infection”, matching the wording performed through the entire manuscript.

Abstract (Lines 20-23): Alcohol consumption and fitness centers were weakly associated with infection, in agreement with findings of our similar study conducted six month earlier. Apart from the two factors, no community activities were more common amongst cases, suggesting that community restrictions in place were adequate. The strongest risk factor for transmission was contact to an infected person.

3. In the abstract, the sentence “For reference, we provide a timeline of non-pharmaceutical interventions in place in Denmark from February 2020 to March 2022” should be in the earlier part of the abstract to flow better.

**Answer**: Thank you for this suggestion, the sentence have been moved to an earlier part of the abstract (lines 12-13). The title of the manuscript have also been update, to include ‘overview of societal COVID-19 epidemic counter measures in Denmark’ for a better flow and understanding.

4. In the abstract, the citation should not be included and should be removed.

**Answer**: The citation has been deleted.

5. If there are any missing data, they should be elaborated on in the tables and provide information on how they were addressed. If not, please ignore this comment.

**Answer**: There was no missing data. In the analysis presented in table 3, all ‘do not know’ responses were excluded (stated in the footnote under the table).

6. This is a suggestion, but authors should reconsider whether there is a need to do the analysis excluding cases who reported to be infected in their household (Table 4) when the authors are doing an analysis excluding individuals with close contact (Table 5)? I understand that the former data provided some factors that are statistically significant while the latter did not, but it seems arbitrary.
 **Answer:** Thank you for this relevant suggestion. The purpose of Table 5 was to see if self-isolation could explain why more controls were often exposed than cases (resulting in OR estimates below 1). Persons who recently had been in close contact with an infected person would self-isolate, as recommended, and would therefore not be having activities in the community (if they had this information during their exposure period!). Removing those with known contact to an infected person did not change the results.

The intension with Table 4, excluding household transmission, is to remove those exposed at home, i.e. those not relevant for assessing risk associated with activities in the community.

We do not have detailed information about the setting of the close contact (those excluded in Table 5), and could thus be excluding transmission occurring during community activities if implementing your suggestion. No changes have been made to the manuscript.

7. The reason to exclude cases who are close contact is partly that cases most likely acquired the virus through this specific contact rather than other community exposures you are asking about in the questionnaire. This can also be mentioned in the manuscript.

**Answer**: Thank you this valid point. We unfortunately do not have detailed information about the setting of the close contact that would allow us to tease out which close contact occurred during community activities and which occurred at home or during non-community activity. We have mentioned this limitation in the manuscript.

Line 283-288 We were able to exclude cases exposed in their household, which allowed us to produce a more specific estimate to risk associated to activities in the community. Unfortunately we did not have detailed enough information about the setting of the close contacts not part of the household, to include only those occurring during activities in the community. This means that we may have included cases which may have been infected during home or during non-community activities, which may have weakened the associations.

Reviewer #2: The manuscript "Societal COVID-19 epidemic counter measures and activities associated with SARSCoV-2 infection in an adult unvaccinated population – a case-control study in Denmark, June 2021" is a valuable contribution. Although it does not providing ground-breaking or surprising results, it adds evidence to a steep learning curve from COVID-19 pandemic for epidemiologists, social scientists and policymakers. This contribution is a good example of robust and rapid evidence for policy that can be immediately applied during the crisis and build evidence to better prepare for future pandemics.

General suggestions on methods:
- Since the outcome of interest is tested positive for SARS-CoV-2, could the authors elaborate on the national criteria for testing that were used during the study period? It is relevant for the international audience, since in every country a "case" could have a slightly different probability of being identified. It is mentioned in the discussion that there was massive testing, possibly including asymptomatic people without any indications, in the occupational setting. In such a case, it is worth introducing this context, and addressing the issue of RT-PCR validity and reliability in the limitation section.
 **Answer**: Thank you for this comment, additional information on the testing recommendations and references have been added to the discussion.

Lines 294-301: In the study period, participation in societal activities generally required regular testing regardless of symptoms as part of the corona passport strategy in our unvaccinated, not previously infected study population. Persons who had had contact with a SARS-CoV-2 infected person or had symptoms of COVID-19 were recommended one or more tests and additionally recommendations or regular screening tests existed for certain professions [28, 29]. During the period around 11,000-26,000 tests were performed per 100,000 population [30] and seroprevalence studies based on blood donors showed that a low proportion the adult healthy population of Denmark had been infected [31]. Therefore, we believe that controls were unlikely to have been positive without knowing.

- Could the authors explain a bit more why they applied individual matching? With such massive exposures and such common infection, it would be perhaps better to use unmatched design and check for the effect of age and sex on the infection risk? The purpose of the control group is to represent exposures in the source population and matching is a way to prevent confounding. If age and sex are not confounders in a given scenario, matching can actually introduce confounding. I recommend adding to the Methods section a short explanation/rationale to apply individual matching.

**Answer**: Thank you for this question, we applied individual matching as we assume that the matching variables were confounders. We assume that age, sex and place of residence affect your behaviour (how often you are dinning at a restaurant, visiting bars, drinking alcohol, going to fitness, but also adherence to official guidelines), number of contacts and the local reproduction rate. No adjustment have been made to the manuscript.

- Could the authors explain the rationale behind excluding residents previously vaccinated and previously tested positive by RT-PCR? We know now that previous infection or vaccination does not prevent new infections. If one wants to investigate the effect of community exposures on disease risk, maybe it would be a better idea to not exclude infections occuring >6 months previously, for example. Maybe the authors just assumed that with a new disease circulating for a short time, it is safe to assume that all were recently vaccinated and all infected to date were "recently" infected and thus were immune to SARS-CoV-2 infections... These assumptions should be however better explained and addressed in the limitations section.

**Answer**: Thank you for this comment. Michlmary et al. 2022, investigated the observed protection against SARS-CoV-2 reinfection following a primary infection. The study was performed in Denmark, in the same period as our study was performed. They showed that SARS-CoV-2 infection and vaccination offered a high level of protection against reinfection. Based on this, we assumed that the risk of infection/reinfection was not the same for those previously SARS-CoV-2 positive, not previous infected and those vaccinated against SARS-CoV-2. In order not to include people with different risk of infection, the study was performed among people who had not previously tested positive. The following adjustment have been made:

Lines 91-92: Only controls unvaccinated and not previously infected by 12 June 2021 were included to match the risk of infection profile of the cases.

Reference: Michlmayr, D., Hansen, C. H., Gubbels, S. M., Valentiner-Branth, P., Bager, P., Obel, N., Drewes, B., Møller, C. H., Møller, F. T., Legarth, R., Mølbak, K., & Ethelberg, S. (2022). Observed protection against SARS-CoV-2 reinfection following a primary infection: A Danish cohort study among unvaccinated using two years of nationwide PCR-test data. The Lancet regional health. Europe, 20, 100452. https://doi.org/10.1016/j.lanepe.2022.100452

- I recommend using the term matched odds ratios (mOR) instead of odds ratios, to reflect the methodological approach. It should be updated in the entire manuscript, including the abstract.

**Answer**: Thank you. We have now specified the type of odds ratios where the specification lacked.

Lines 9-11: We determined matched odds ratios (mORs) and 95% confidence intervals (95%CIs) by conditional logistical regression with adjustment for household size and immigration status.

Lines 15-19: Reporting close contact with an infected person who either had or did not have symptoms resulted in mORs of 20 (95%CI:9.8-39) and 8.5 (95%CI 4.5-16) respectively. In contrast, community exposures were generally not associated with disease; several exposures were negatively associated. Exceptions were: attending fitness centers, mOR=1.4 (95%CI:1.0-2.0) and consumption of alcohol in restaurants or cafés, aOR=2.3 (95%CI:1.3–4.2).

- As mentioned in previous comments, the study has several limitations, not thoroughly revised by the authors. I recommend to be a bit more comprehensive in this matter!

**Answer**: Thank you for this recommendation. In order to accommodate, the section in the discussion covering the methodological strengths and limitation have been revised. The section now includes concerns about recall bias and parts have been rephrased to improve readability.

Lines 267-301: Methodological strengths and limitation outlined in our previous 2020-study also apply for the current study. Among the limitations of the first study was the small sample size, therefore we went from 600 to 1000 participants to strengthen the power of the present study. We did not find any high mOR with wide confidence intervals, indicating type I errors, and therefore we consider our sample size to have been sufficient to identify associations had they existed. Compared to our first study, we also shortened the exposure period inquired about, aiming to provide more specific estimates of associations. The use of the Danish Vaccination Registry enabled us to swiftly and objectively exclude those who had been vaccinated by the time of the study and used the Danish Microbiology Database to exclude those previously infected.

A potential bias would arise from systematic differences in behavior between cases and controls. Persons who recently had been in close contact with a person with SARS-CoV-2 infection, would, if they were aware of the exposure at the time, likely have been in self-isolation and would therefore not be active in the community. Because we for many activities found controls to be more exposed than cases (resulting in OR estimates below 1), we were suspicious of such a bias being at play. To explore this further, we performed a sensitivity analysis, in which we excluded all participants who reported to have been close contacts to infected persons. This did not change the results. We were able to exclude cases exposed in their household, which allowed us to produce a more specific estimate to risk associated to activities in the community. Unfortunately we did not have detailed enough information about the setting of the close contacts not part of the household, to include only those occurring during activities in the community. This means that we may have included cases which may have been infected at home or during non-community activities, which may have weakened the associations. Another potential systematic difference between cases and controls is their ability to recall activities in the period in question, if cases already during contact tracing had been asked to recall relevant activities, they may have been more likely to report such activities adequately, which could have influenced the results. Another potential concern relates to the selection of controls. We used matched controls sampled from the general population, which was made possible because of our access to the Danish Civil Registration System. A different approach, which we did not opt for, would have been control selection with recruitment from the pool of persons testing negative in PCR test in the same period as the pool of cases tested positive. This option has been used by others, and we cannot say how it would have influenced on our results [5, 9]. In the study period, participation in societal activities generally required regular testing regardless of symptoms as part of the corona passport strategy in our unvaccinated, not previously infected study population. Persons who had had contact with a SARS-CoV-2 infected person or had symptoms of COVID-19 were recommended one or more tests and additionally recommendations or regular screening tests existed for certain professions [28, 29]. During the period around 11,000-26,000 tests were performed per 100,000 population [30] and seroprevalence studies based on blood donors showed that a low proportion the adult healthy population of Denmark had been infected [31]. Therefore, we believe that controls were unlikely to have been positive without knowing.

Minor corrections:
- Abstract line 10: Please move the numbers to the results
**Answer**: The number of included cases and controls have been removed to the results.

- Abstract lines 20-21: Do not include citations in the abstract.
**Answer**: The reference have been deleted from the abstract.

- Introduction line 39: Why to refer to case-control studies? Do authors refer to studies in Denmark? It would be good to be more precise...
**Answer**: The sentence have been updated.
Line 44: By use of a case-control design, researchers all over the world have aimed to identify determinants, private and societal, for SARS-CoV-2 infection.

- Methods line 70: typo in "ministries".
**Answer**: Thank you for noticing this. It has been corrected.
Line 75-76: The information was retrieved from relevant Danish government ministries and from the national COVID-19 communication partnership (coronasmitte.dk).

- Results line 163: did the authors mean: "Compared to eligible cases, eligible controls [...]"?
**Answer**: Thank you for making us aware that this sentence might be misunderstood. We refer to the difference between eligible cases (unvaccinated individuals between 18–49 years old, with an address in Denmark, and an RT-PCR confirmed SARS-CoV-2 infection in the period from 8 to 12 June 2021) and included cases in the study. We have made minor adjustments to the manuscript to make the point clearer.

Lines 174-175: Compared to eligible cases diagnosed during the period, the recruited cases were less likely to have migrant background (data not shown).

- Discussion 279: "conducting" not "conduction"
**Answer**: thanks, the spelling mistake have been corrected.
Lines 298-303: Since no determinants for infection were identified, we conclude that overall societal restrictions in use in the spring of 2021 were adequate to reducing any potential risk associated with community activities such as participating in cultural events, dining at restaurants, shopping and public transportation; exceptions being fitness centers and alcohol consumption. Instead, a major risk factor for transmission was contact to an infected person and transmission primarily took place via infected colleagues or family members.

Reviewer #3: This is a case-control study attempting to identify individual and contextual risk factors for SARS-CoV-2 infection in Denmark. The study is well written and has a sound methodology. However, I have a few questions that I believe should be addressed:

- In the abstract (line 4) the authors mention they aim at testing the efficiency of such measures - however efficiency requires a resources dimension - do the authors believe they have addressed this?

**Answer**: Thank you for this comment. We have revised the first part of the abstract and the conclusion so that it is more clear.

Lines 2-4: Measures to restrict physical inter-personal contact in the community have been widely implemented during the COVID-19 pandemic. We studied determinants for infection with SARS-CoV-2 with the aim of informing future public health measures.

Lines 20-23: Alcohol consumption and fitness centers were weakly associated with infection, in agreement with findings of our similar study conducted six month earlier. Apart from the two factors, no community activities were more common amongst cases, suggesting that community restrictions in place were adequate. The strongest risk factor for transmission was contact to an infected person.

- At the end of introduction (lines 60-61) authors mention the "present an overview of the official restrictions". This seems a bit misleading as the authors actually prepare one as part of their work. I suggest this to be rephrased.

**Answer**: The sentence have been rephrased.
Lines 59-61: Here we present the results of a second national case-control study of risk factors for infection. For context and reference, we further created an overview of the official restrictions that have been in place in Denmark throughout the COVID-19 epidemic.

- Authors have excluded hospitalized and vaccinated patients. Can they comment on how this might have influenced the results and how this can affect results generalizability?

**Answer**: .We studied determinants for infection with SARS-CoV-2 and were primarily interested in community exposures, and therefore we excluded cases and controls who had been hospitalized for more than 12 hours in the period of interest.

Cases and controls who have been vaccinated before the 8 of June 2021 was also excluded. Michlmary et al. 2022, investigated the observed protection against SARS-CoV-2 reinfection following a primary infection. The study was performed in Denmark, in the same period as our study was performed. They showed that SARS-CoV-2 infection and vaccination offered a high level of protection against reinfection. Based on this, we assumed that the risk of infection was not the same for vaccinated and non-vaccinated persons. In order not to include people with different risk of infection, the study was performed among people who had not previously tested positive. That the study population were unvaccinated not previously infected persons limits the generalizability to the vaccinated – at the time older – population. Conclusions generalizable to the vaccinated population would have required a different design, for example with two or more case and control groups. No adjustment have been made to the manuscript.

Reference: Michlmayr, D., Hansen, C. H., Gubbels, S. M., Valentiner-Branth, P., Bager, P., Obel, N., Drewes, B., Møller, C. H., Møller, F. T., Legarth, R., Mølbak, K., & Ethelberg, S. (2022). Observed protection against SARS-CoV-2 reinfection following a primary infection: A Danish cohort study among unvaccinated using two years of nationwide PCR-test data. The Lancet regional health. Europe, 20, 100452. doi:10.1016/j.lanepe.2022.100452

- Data collection: The exposure period ranged from eight to two days before the symptom onset (or test for asymptomatic) for cases. However I could not identify what was the index date for controls - what was the 6-day refer to? Can the authors clarify?

**Answer**: The 6-day period for controls, were the same as for their matched case. We have made minor adjustments to the text, and hope it is clearer now:

Lines 113-114: The 6-day period ran from eight to two days prior to symptom onset (or test date if asymptomatic) for cases and the same 6-day period for the matched control.

- Some of the analysis reported in Table 1 and not described in the methods. I suggest this to be added.
**Answer**: Thank you for this suggestion, the methods section have been updated accordingly.

Lines 139-141: We compared basic demographic characteristics (country of origin, household size, number of contacts and employment status) of cases and controls. We used Chi-Square to test for overall differences and matched logistic regression to test for intergroup differences.

- The authors emphasize the alcohol consumption in their results. However this has not been given an in-depth consideration in the discussion. Why the authors believe they have identified this results? Is it really related with the alcohol consumption or it this a proxy for other types of behaviours?

**Answer**: Thanks for this point. The following have been added to the discussion:

Lines 229-237: As also speculated previously [10], activities potentially involving heavy breathing, excretions of aerosols, close interaction and touching of multiple surfaces in a closed indoor environment could constitute an environment prone to SARS-CoV-2 transmission, compared to outdoor sport activities. In the same line is the association with alcohol when present at restaurants or cafés, where alcohol may be a proxy for reduced awareness of protective behavior and adherence to IPC recommendations, increasing the likelihood of closer interactions and with that SARS-CoV-2 transmission. On the other hand, many other investigated activities, notably use of public transportation, supermarkets and cultural and sports gatherings were as common in cases than amongst controls.

- There are negative results in for a series of contexts. Have the authors explored these results in more depth? They raise an hypothesis is the discussion (lines 261 onwards) which has not been corroborated by the sensitivity analysis. Do you see any alternative reasons for these results. Could remote work play a role here?

**Answer**: Recall bias have been added as a potential systematic bias between cases and controls. However, it has not been possible to investigate this potential bias. Unfortunately we do not have information on remote work.

Lines 280-283: Another potential systematic difference between cases and controls is their ability to recall activities in the period in question, if cases already during contact tracing had been asked to recall relevant activities, they may have been more likely to report adequately, which can have influenced the results.

- The authors claim their results support the "efficiency" of existing measures. However I am not entirely convinced here. In fact there is no counterfactual to assess the effectiveness (which I believe is what in fact authors intended to assess) or the impact of those measures. Please comment.

**Answer**: We have updated the main text and conclusion to make the message we wanted to convey clearer. We interpret our finding that we, with the exception of alcohol and fitness, did not identify any risk associated with community activities, as that the measures in place were adequate to reduce any potential risk to a level where we could not find a difference between cases and controls. Yes, transmission have happened in those settings, but not to an extent where we would be able to predict where such a transmission would be more likely to occur. Contact to an infected person was, not surprisingly, by far the strongest risk factor for infection. So is state that as we found no single determination to be associated with SARS-CoV-2 infection and because transmission appeared to primarily take place via contact to a person with known SARS-CoV-2 infection, community restrictions in place were efficient.

Abstract (Lines 20-23): Alcohol consumption and fitness centers were weakly associated with infection, in agreement with findings of our similar study conducted six month earlier. Apart from the two factors, no community activities were more common amongst cases, suggesting that community restrictions in place were adequate. The strongest risk factor for transmission was contact to an infected person.

Conclusion (Lines 296-304): In conclusion, we show results of a study of risk factors for SARS-CoV-2 infection and compare with a similar study done six month earlier. Under the constraints of the methodology of case-control studies, no major determinants for infection were identified. Since no determinants for infection were identified, we conclude that overall societal restrictions in use in the spring of 2021 were adequate to reducing any potential risk associated with community activities such as participating in cultural events, dining at restaurants, shopping and public transportation; exceptions being fitness centers and alcohol consumption. Instead, a major risk factor for transmission was contact to an infected person and transmission primarily took place via infected colleagues or family members. Finally, we provide a timeline of non-pharmaceutical interventions that were implemented in Denmark from February 2020 to March 2022.

Reviewer #4: The authors aim to identify determinants of Sars-COV2 infection in the Denmark in June 2021 using a case –control study. They compare exposure to determinants among
1) 500 cases unvaccinated; 18-49 years old, residents in Denmark, with a RT-PCR test positive result between 8-12 June that were not hospitalized; had not travelled abroad
2) With 529 controls matched on year of birth, sex and municipality with the cases (1 to 1 match).
The 6 days (from Day -8 to Day -2 prior to symptom or test +) exposure to determinants was measures by telephone interviews between 15 and 24 June. Conditional logistic regression is correctly use for the analysis.

- The population from which cases and controls are coming from does not look identical concerning previous infection which is an exclusion criteria for controls but no clear info is provided about the cases. For the community transmission, given the restrictions in place, it means that we could have only controls with a recent negative test but we could have cases with a previous infection or a recent negative test who access public spaces. A subgroup analysis could be performed excluding cases with previous infection and their matched controls, to account for that.

**Answer**: Thank you for this comment. We apologise that it was not clearly described in the methods, but cases previously infected by 8 June 2021 were not included. Meaning that, cases and controls was similar concerning previous infection status. This have been added to the methods and adjusted in the abstract.

Lines 5-6: We conducted a national matched case-control study among unvaccinated not previously infected adults aged 18-49 years

Lines 89-91: Eligible cases were unvaccinated individuals between 18–49 years old, not previously infected by 8 June 2021, with an address in Denmark, and an RT-PCR confirmed SARS-CoV-2 infection in the period from 8 to 12 June 2021.

- In order to understand the results without the need to consult other articles a more detailed description of the questionnaire used is necessary.

**Answer**: Thank you for this suggestion. A description of the included community exposures have been included.

Lines 122-126: The community exposures inquired about were same as in our first study, and included activities as dining at restaurants, going to bars, shopping, participating in sport activities, and religious events or events involving singing etc. along with questions on if these activities took place indoors or outdoors, or involved consumption of alcohol. In contrast to the first study, we did not include questions on protective behavior and adherence with measures.

-The conclusion that Contact with another individual with a known infection as the main determinant for SARS-CoV-2 infection is based on sound methodology and nicely reflected in the different sections. Given the strength of the association even adjusting for other determinants will not modify the findings and or conclusions.

Sub-group analysis for the community transmission section.
The analysis for each determinant is performed separately adjusted for migration back ground, household size and the matching variables. There is no analysis performed by adjusting for all determinants, or for determinants associated with risk of infection in the bivariate analysis.

**Answer:** Thank you for this comment. As we do not identify any strong associations, data does not give rise to additional analyses. We fear that any multivariable (apart from the confounding factors adjusted for) analyses will strain our data to a point not meaningful.

- Based on the results from table 4 some of the interpretations about differences between cases and controls are not fully correct. In the results section it states that cases are more likely than controls to have consumed alcohol in a bar. By looking at table 4, the CI95% of the OR for alcohol consumption in a Bar includes the 1 meaning that the difference is not statistically significant. The same logic applies for the indoor sport activities, indoor fitness center and outdoor sport activities. The difference between cases and controls is not statistically significant. The results and discussion sections are to be updated accordingly concerning the community transmission part. (no significant difference for indoor fitness centers, indoor sport activities, alcohol consumption in bars )

**Answer**: Thank you for this point. We have added that the trends we found, that more cases than controls consume alcohol at bar and indoor cultural events are not significant.

Lines 183-188: Controls were more likely to report having been to restaurants than cases, mOR: 0.66 (95% CI: 0.49-0.90). However, cases were more likely than controls to report, that they or others in their company had consumed alcohol during the restaurant visit, adjusted odds ratio (aOR): 2.3 (95% CI: 1.3-4.2). The same trend was seen for bar and indoor cultural events, where more controls than cases reported to have visited a bar and participated in indoor cultural events, but more cases than controls reported alcohol consumption (non-significant) (Table 4).

- It seems that the study is underpowered to detect community determinants of SARS-COV2 infection. Considering a matched 2 controls 1 case design could partially resolve that problem. In the multivariate model, only determinants associated with outcome in the bivariate analysis could be entered.

**Answer**: Thank you for this comment. We did not find any high mOR with wide confidence intervals (except within the sensitivity analysis), which could be a sign of a type I error, and we therefore believe the sample size would be sufficient to identify associations if they existed. Also, we had beforehand performed a power estimation; this is described in the MS. We have added a comment on this in the limitation section.

We did not find several community exposures to be associated with SARS-CoV-2, and therefore we did not find a multivariate model relevant.

Lines 268-270: We did not find any high mOR with wide confidence intervals, indicating type I errors, and therefore we consider out sample size to be sufficient to identify associations if they existed.

- Other
Page 4 line 56 (Introduction section) Please rephrase
Society was gradually reopening, with now only societal restrictions in place for those individuals who were vaccinated, had recovered from infection or recently tested negative
In contradiction with Line 71-72 (methods section)

**Answer**: Thank you for this comment. The introduction have been corrected.

Lines 56-58: Society was gradually reopening, with now only societal restrictions in place for those individuals who were not vaccinated or protected from previous infection and for those who did not have a recently negative SARS-CoV-2 test.

- Table 1 Please provide OR for non matching variables. From the bivariate analysis it looks like the migration background and the number of contacts are associated with the risk of becoming a case.

**Answer:** Table 1 have been updated and now the table includes the p-value for the Chi-Square test for overall difference between cases and controls, as well the p-values for the matched odds ratios have been included. The methods section have also been updated

Lines 139-141: We compared basic demographic characteristics (country of origin, household size, number of contacts and employment status) of cases and controls. We used Chi-Square to test for overall differences and matched logistic regression to test for intergroup differences.

| **Table 1. Number and proportion of included cases and controls by demographic characteristics and p-value of test for deviations, Denmark, June 2021.** | | | |
| --- | --- | --- | --- |
| **Demographic characteristics** | **Included cases (n=500), n (%)** | **Matched controls (n=529), n (%)** | ***P* value^1^** |
| *Age group* |  |  | na |
| 18-24 years | 199 (40) | 212 (40) |  |
| 25-34 years | 159 (32) | 170 (32) |  |
| 35-44 years | 85 (17) | 88 (17) |  |
| 45-49 years | 57 (11) | 59 (11) |  |
| *Sex* |  |  | na |
| Male | 263 (53) | 283 (54) |  |
| Female | 237 (47) | 246 (47) |  |
| *Region* |  |  | na |
| Capital Region of Denmark | 195 (39) | 200 (38) |  |
| Region Zealand | 56 (11) | 62 (12) |  |
| Region of Southern Denmark | 61 (12) | 66 (12) |  |
| Central Denmark Region | 117 (23) | 128 (24) |  |
| North Denmark Region | 71 (14) | 73 (14) |  |
| *Migration background^2^* |  |  | *<0.05* |
| Denmark | 403 (81) | 462 (87) | Ref. |
| Western country | 26 (5.2) | 23 (4.3) | 0.34 |
| Non-western country | 71 (14) | 44 (8.3) | <0.05 |
| *Household size^3^* |  |  | *0.64* |
| 1 | 78 (16) | 91 (17) | Ref. |
| 2 | 141 (28) | 165 (31) | 0.92 |
| 3 | 109 (22) | 100 (19) | 0.22 |
| 4 | 105 (21) | 108 (20) | 0.46 |
| ≥5 | 67 (13) | 65 (12) | 0.39 |
| *Number of contacts* |  |  | *<0.05* |
| 0-5 | 290 (58) | 208 (39) | Ref. |
| 6-10 | 88 (18) | 156 (29) | <0.05 |
| 11-15 | 47 (9.4) | 51 (9.6) | 0.06 |
| 16-20 | 27 (5.4) | 30 (5.7) | 0.07 |
| 21-49 | 30 (6.0) | 52 (9.8) | <0.05 |
| Over 50 | 18 (3.6) | 32 (6.0) | <0.05 |
| *Employment status* |  |  | *0.34* |
| Employed | 307 (61) | 339 (64) | Ref. |
| Student | 150 (30) | 138 (26) | 0.06 |
| Other | 43 (8.6) | 52 (9.8) | 0.62 |

^1^ P-value for Chi-Square test for deviation between included cases and matched controls (italic). P-value for mOR (non-italic)
^2^ Non-Danish migration background was defined as first or second generation immigrants from either Western countries (primarily neighboring European countries) or from non-Western countries (the five most frequent being Turkey, Iraq, Lebanon, Pakistan, and Somalia).
^3^ Number of registered persons on the same address*.*

In table 2 there is a total of 516 cases reported whereas in the results section only 500 cases are reported as included, please double-check or clarify

**Answer**: Thank you for noting this.
In table 2, 500 cases were included and the 500 cases indicated 516 likely places of infection. The denominator in the table have been changed to 500 instead of 516. This is stated under the table. Additional, the ^1^, have been moved to the title, so make it more clear.

| **Table 2. Number and proportion of likely place of infection as indicated by cases (n=500)^1^.** | |
| --- | --- |
| **Likely place of infection** | **n (%)** |
| Household | 105 (21) |
| Friends/other family than household | 80 (16) |
| Workplace | 84 (17) |
| Education | 28 (5.6) |
| Leisure activities | 27 (5.4) |
| Other events | 13 (2.6) |
| Public transport | 8 (1.6) |
| Other place/exposure | 68 (14) |
| Don’t know | 103 (21) |

^1^It was possible to indicate more than one likely place of infection (516 answers included from 500 cases).

Table 3 Please provide adjusted OR for migration background and household size as well.

**Answer:** Thank you for this suggestion, we have added the OR for migration and household size from the type of contact model in table 3:

Table 3:

**Table 3. Number, proportion and matched odds ratios (mOR) related to type of contact with a person with known SARS-CoV-2 infection (with/without symptoms), Denmark, June 2021.**

| ***Type of contact with infected person with/without symptoms*** | **Cases (n=483), n (%)** | **Controls (n=512), n (%)** | **mOR (95% CI)** |
| --- | --- | --- | --- |
| No contact with infected person | 256 (53) | 472 (92) | Ref. |
| *Other contact,* without symptoms | 10 (2.1) | 6 (1.2) | 3.04 (0.93-9.99) |
| *Other contact,* with symptoms | 14 (2.9) | 6 (1.2) | 3.25 (1.15-9.19) |
| *Close contact,* without symptoms | 73 (15) | 16 (3.1) | 8.53 (4.52-16.11) |
| *Close contact,* with symptoms | 130 (27) | 12 (2.3) | 20 (9.80-39.49) |
| *Migration background* |  |  |  |
| Denmark | 391 (81) | 448 (88) | Ref. |
| Western country | 25 (5.2) | 23 (4.5) | 1.13 (0.52-2.43) |
| Non-western country | 67 (14) | 41 (8.0) | 2.17 (1.28-3.68) |
| *Household size* |  |  |  |
| *1* | 75 (16) | 86 (17) | Ref. |
| *2* | 135 (28) | 159 (31) | 0.93 (0.56-1.54) |
| *3* | 105 (22) | 98 (19) | 1.07 (0.63-1.79) |
| *4* | 102 (21) | 106 (21) | 1.25 (0.72-2.18) |
| ≥5 | 66 (14) | 63 (12) | 0.88 (0.47-1.64) |

Note mOR estimates were adjusted for type of contact with/without symptoms, migration background and household size.
All ‘do not know’ responds were excluded in the analysis.

General Feedback
Please standardize the reporting of OR and CI95% by using everywhere 2 decimals (1,01 instead of 1,0 or 39,00 instead of 39).
Inclusive tables with absolute numbers and proportions as well as OR(CI95%) of both bivariate and multivariate analysis would be more easy to read and interpret.

**Answer:** Thank you for this comment. We would be sad to report more than two significant digits on percentages and in the text, as we believe having only two significant digits improves readability and does not compromise the interpretation of the results. If the editor of PLOS ONE insist, we will of course adjust.

**Additional revisions:**

Cajar et al., 2022 have been published meanwhile, so the reference have been updated.

Lines 255-259: Another Danish case-control study, performed in October to December 2020 found associations similar to those seen in our studies [5]. The most important risk factor identified was contact to an infected person. Moreover, this study also found an association with fitness centers but not with shopping, use of public transport and participating in outdoor sport activities. Contrary to our findings, participation in indoor sport activities, larger events, and restaurant and bar visits were identified as risk factors [5]

Reference: Cajar MD, Tan FCC, Boisen MK, Krog SM, Nolsoee R, Collatz Christensen H, et al. Behavioral factors associated with SARS-CoV-2 infection. Results from a web-based case-control survey in the Capital Region of Denmark. BMJ Open. 2022;12(6):e056393. doi: 10.1136/bmjopen-2021-056393

The link in reference [11] have been updated.

Reference: The Danish Ministry of Health. Orientering om ændringer i regler vedr. coronapasset pr. 21. maj 2021. https://sum.dk/Media/637571404984193343/Orientering%20om%20%C3%A6ndringer%20i%20regler%20vedr.%20coronapas%20pr.%20215.pdf.

6. PLOS authors have the option to publish the peer review history of their article ([what does this mean?](https://journals.plos.org/plosone/s/editorial-and-peer-review-process#loc-peer-review-history)). If published, this will include your full peer review and any attached files.

If you choose “no”, your identity will remain anonymous but your review may still be made public.


**Do you want your identity to be public for this peer review?** For information about this choice, including consent withdrawal, please see our [Privacy Policy](https://www.plos.org/privacy-policy).

Reviewer #1: No

Reviewer #2: **Yes:** Pawel Stefanoff

Reviewer #3: **Yes:** Andreia Leite

Reviewer #4: No

[NOTE: If reviewer comments were submitted as an attachment file, they will be attached to this email and accessible via the submission site. Please log into your account, locate the manuscript record, and check for the action link "View Attachments". If this link does not appear, there are no attachment files.]

While revising your submission, please upload your figure files to the Preflight Analysis and Conversion Engine (PACE) digital diagnostic tool, <https://pacev2.apexcovantage.com/>. PACE helps ensure that figures meet PLOS requirements. To use PACE, you must first register as a user. Registration is free. Then, login and navigate to the UPLOAD tab, where you will find detailed instructions on how to use the tool. If you encounter any issues or have any questions when using PACE, please email PLOS at [figures@plos.org](mailto:figures@plos.org). Please note that Supporting Information files do not need this step.
