# Supplementary material for: Case-control study of activities associated with SARS-CoV-2 infection in an adult unvaccinated population and overview of societal COVID-19 epidemic counter measures in Denmark
Source: PLoS One. 2022 Nov 16;17(11):e0268849. doi: 10.1371/journal.pone.0268849 (PMC9668151; doi:10.1371/journal.pone.0268849)
Supplement: Attachment — Submitted filename: Response to Reviewers.docx [file pone.0268849.s002.docx]

**Rebuttal letter, 'Response to Reviewers'. Revision no 2.**

PONE-D-22-13314R1

Case-control study of activities associated with SARS-CoV-2 infection in an adult unvaccinated population and overview of societal COVID-19 epidemic counter measures in Denmark

*Changes to the reference list are mentioned at the end of this doc.*

**Reviewer #1:** The authors addressed most of my comments in a sufficient manner. However, I am still not convinced about the validity of their conclusion, which is critical for this paper. As I mentioned before, even if the community restrictions in place were efficient, behaviors that are truly high-risk would have been identified as such in the present study design. For example, individuals who were positive would be more likely to have history of going to bars and restaurant (resulting in higher odds) if this specific activity is indeed a high-risk behavior in the context of the study population. This should be the case regardless of what type of community restriction is in place. Conversely, as observed in this study, if we do not see any association between a specific behavior and infection, then it would not be possible to conclude that “community restrictions in place were efficient” as authors have done. If history of going to bars and restaurant is really high-risk, then we would see higher ORs. If the policy implemented includes restricting the opening hours at bars and restaurants and if we see that there is a good association between going to bars and restaurants, then we can infer that the policy is appropriately targeting high-risk individuals and high-risk behaviors. Below paper illustrated this exact point: Behavioral factors associated with SARS-CoV-2 infection in Japan. Influenza Other Respir Viruses. 2022;16(5):952-961.

**Author reply:** Thank you for raising this important point. We only partly agree. The study was done while restrictions concerning eg restaurants and bars were in place. They for instance involved restricted opening times, restrictions in the number of patrons present and use of masks. It is possible that we’d have seen restaurants appear as risk factors in a (hypothetical) un-restricted scenario but that the risk was lowered by the restrictions in place, resulting in no excess risk (measured as an odds ratio here) being identified. It is true of course that we cannot know this. It is also possible that the restrictions were redundant or simply had no effect and that we’d have obtained identical results had they not been in place. For this reasons we previously used to word ‘suggesting’.

However, we would agree that we might have stretched our conclusion too far and have therefore now changed the wording in the abstract and in the last conclusion paragraph of the MS. We now no longer say that the restrictions may have been adequate, rather that we cannot know this.

In the Abstract, the lines “Apart from these two factors, no community activities were more common amongst cases, suggesting that community restrictions in place were adequate.”

Have now been changed to: “Apart from these two factors, no community activities were more common amongst cases under the community restrictions in place during the study” (lines 18-19).

In the Conclusion, the revised text now reads:

“We conclude that transmission in the general community was of little importance, while instead, the major risk factor for transmission was contact to a known infected person and that transmission primarily took place via infected colleagues or family members. Our study could not directly measure the effect of the societal restrictions in place but it is not unreasonable to expect that these had an effect in reducing any potential risks associated with community activities such as participating in cultural events, dining at restaurants, shopping and public transportation.” (lines 307-312).

**Reviewer #2:** The authors have carefully considered all comments of reviewers. Therefore, I recommend to accept in the current form.

**Author reply:** Thank you. No changes made to the manuscript.

**Reviewer #3:** Many thanks for fully considering the comments submitted and having provided satisfactory replies. While my comments were fully addressed I have 2 questions from replies from other reviewers:

- Authors have mentioned type I errors while refering to issues with power (R#2 and R#4). However I believe they intended to refer to type II erros (failure to reject a null hypothesis when it is false). Please review.

**Author reply:** Thank for you noticing this mistake. We have now rephrased the section:

Line 273: We did not find any high mOR with wide confidence intervals, which could indicating risk of type II errors, and therefore we consider our sample size to have been sufficient to identify associations had they existed

- Authors have mentioned in response to R#2 - "During the period around 11,000-26,000 tests were performed per 100,000 population [30]". I suggest to clarify whether these include only PCR or also Rapid Antigen Tests.

**Author reply:** Thank you for this comment. The number refers to PCR tests. This have now been added to the manuscript.

Lines 300-301: During the period around 11,000-26,000 RT-PCR tests were performed per 100,000 population

**Reviewer #4:** The majority of the comments have been addressed. However there are still few remaining issues to be addressed

- General Comment :
Please consistently report two decimals for OR CI95% throughout the text.

**Author reply:** Thanks for this. We’ve tried to consistently report two significant figures for OR’s and CI’s. Reporting two decimals would not be correct as it would imply precision in measurements that is not present. We for instance report “mOR: 20 (95% CI: 9.8-40)”. Here the number of significant figures are two for all three reported numbers. It would not have been correct to report, eg: “mOR: 19.89 (95% CI: 9.81-40.01), or [as a more extreme example]: “mOR: 163,12 (95% CI: 9.81-1936.23)”. (Please, if interested, see also: <https://en.wikipedia.org/wiki/Significant_figures>). We note that reporting of decimals is a matter of precision but also journal style and that the journal will decide given acceptance of the MS.

- In the table 4 for in indoor sport activities, mOR: 0.57 (95% CI: 0.32-1.01), and for outdoor sport activities mOR: 0.71 (95% CI: 0.49-1.03) does not support the conclusion that controls were more likely to participate in indoor sport and outdoor sport activities.

**Author reply:** Thank you for this comment. We have now have been rephrased the following four sections:

Lines 17-19: “Consumption of alcohol in restaurants or cafés, aOR=2.3 (95%CI:1.3–4.2) and possibly attending fitness centers, mOR=1.4 (95%CI:1.0-2.0) were weakly associated with SARS-CoV-2 infection, in agreement with findings of our similar study conducted six month earlier. Apart from these two factors, no community activities were more common amongst cases, suggesting that community restrictions in place were adequate. The strongest risk factor for transmission was contact to an infected person.”

Lines 192-195: “In total, 24% of cases and 19% of controls had used a fitness centre at least once during the 6-day exposure period, mOR: 1.40 (95% CI: 0.98-2.0), this trend was not seen for indoor sport activities, mOR: 0.57 (95% CI: 0.32-1.0), nor for outdoor sport activities, mOR: 0.71 (95% CI: 0.49-1.0).”

Lines 232-238: “This second study may therefore be seen as corroborating the findings of the first and it would appear that besides direct contact with infected individuals, under the restrictive measures in place in Denmark, social activity involving alcohol and possibly the use of fitness centers constituted actual risk factors. As also speculated previously [10], activities potentially involving heavy breathing, excretions of aerosols, close interaction and touching of multiple surfaces in a closed indoor environment could constitute an environment prone to SARS-CoV-2 transmission, compared to outdoor sport activities.”

Lines 309-314: “We therefore conclude that overall societal restrictions in use in the spring of 2021 were adequate to reducing any potential risk associated with community activities such as participating in cultural events, dining at restaurants, shopping and public transportation; exceptions being alcohol consumption and possibly use of fitness centers. Instead, a major risk factor for transmission was contact to an infected person and transmission primarily took place via infected colleagues or family members.”

- Discussion:
Lines 247-252
The pattern of risk factors seen in the current study was remarkably similar to what we found in our previous study, where also, apart from contact to infected persons with known infections, fitness centers and alcohol consumption in bars and in addition participation in events which involved singing, were identified as being associated with SARS-CoV-2 infection. This second study may therefore be seen as corroborating the findings of the first and it would appear that besides direct contact with infected individuals, under the restrictive measures in place in Denmark, use of fitness centers and social activity involving alcohol constituted actual risk factors.

Comment: Use of fitness centers and participation in events which involved singing are not significantly related to the risk of infection in the second study. Please rephrase.

**Author reply:** Thank you for this comment. We have modified the text accordingly:

Lines 228-235: “The pattern of risk factors seen in the current study was remarkably similar to what we found in our previous study, where also, apart from contact to infected persons, fitness centers and alcohol consumption in bars and in addition participation in events which involved singing, were identified as being associated with SARS-CoV-2 infection. This second study may therefore be seen as corroborating the findings of the first and it would appear that besides direct contact with infected individuals, under the restrictive measures in place in Denmark, social activity involving alcohol and possibly the use of fitness centers constituted actual risk factors.”

- Lines 334 -338
We therefore conclude that overall societal restrictions in use in the spring of 2021 were in fact adequate to reducing any potential risk associated with community activities such as participating in cultural events, dining at restaurants, shoppings and public transportation; exceptions being fitness centers and alcohol consumption. which did constitute risks.

Comment: Use of fitness center is not significantly related to the risk of infection in the second study. Please rephrase.

**Author reply:** Thank you for this comment. We have now modified the text accordingly:

Lines 309-312: “We therefore conclude that overall societal restrictions in use in the spring of 2021 were adequate to reducing any potential risk associated with community activities such as participating in cultural events, dining at restaurants, shopping and public transportation; exceptions being alcohol consumption and possibly use of fitness centers.”

Author: This concludes our responses to the reviewer comments. Please not that in addition we have also made changes, as per Journal Requirements, to the reference list:

**References:**

**The following changes were made to the reference list:**

**Ref. 12**: The link in the reference list have been updated, as the original link wasn’t online anymore.

The updated reference is:

The Danish Ministry of Health Ministry of Industry Business and Financial Affairs and The Ministry of Employment. Retningslinjer om ansvarlig indretning af liberale serviceerhverv og køreskoler i lyset af udbruddet af COVID-19. <https://em.dk/media/14213/retningslinjer-for-liberale-serviceerhverv-af-14-juni.pdf>

**Ref. 20**: The link in the reference list have been updated, as the original link wasn’t online anymore.

The updated reference is:

Aftale om yderligere genåbning pr. 21 maj 2021

<https://coronasmitte.dk/nyt-fra-myndighederne/pressemoeder/aftale-om-yderligere-genaabning>

**Ref 23**: The link in the reference has been deleted.

**Ref 30**: The link to Statens Serum Instituts dashboard has been updated.
